# Older Adults' Perceptions and Use of Patient Portals: A Comparative Analysis of Two Samples

**DOI:** 10.1093/geroni/igab046.3044

**Published:** 2021-12-17

**Authors:** Hyojin Son, Eun-Shim Nahm, Shijun Zhu, Elizabeth Galik, Barbara Van de Castle, Kristin Seidl, Vince Russomanno

**Affiliations:** 1 National Institutes of Health Clinical Center, Bethesda, Maryland, United States; 2 University of Maryland, Baltimore, Maryland, United States; 3 University of Maryland School of Nursing, Baltimore, Maryland, United States; 4 University of Maryland Medical System, Linthicum Heights, Maryland, United States

## Abstract

Older adults can benefit from using patient portals. Little is known whether the perceptions and use of patient portals differ among diverse older adult populations. The aim of this study was to assess the difference in perceived usability of patient portals, self-efficacy for using patient portals, and patient portal use between two adult samples aged 65 years or older. One sample was recruited from a health care system, including hospitals and clinics (n = 174), and the other sample was recruited from nationwide communities (n = 126). Conducting a secondary data analysis using two survey datasets, this study performed a series of linear and ordinal logistic regression analyses. The health care system sample had a higher mean number of chronic diseases and proportion of recent hospitalization than the community sample. The health care system sample showed higher perceived usability, self-efficacy, and usage frequency of patient portals compared to the community sample. eHealth literacy was a significant predictor of perceived usability and self-efficacy. Perceived usability was another significant predictor of self-efficacy. Self-efficacy and health condition variables significantly predicted the more frequent use of patient portals. Compared to the health care system sample, the relationship between perceived usability and use of patient portals was stronger and significant in the community sample. These findings suggest that approaches for promoting patient portal use should consider personal characteristics and health conditions of diverse older adult populations. Future research needs to focus on assessing the impact of using patient portals on older adults’ health care outcomes.

